# The Value of Adding Red Cell Distribution Width to Mehran Risk Score to Predict Contrast-induced Acute Kidney Injury in Patients with Acute Coronary Syndrome

**DOI:** 10.7759/cureus.2911

**Published:** 2018-07-02

**Authors:** Sherif Elhosseiny, Tamer Akel, Jad Mroue, Praveena Tathineni, Suzanne El Sayegh, James Lafferty

**Affiliations:** 1 Internal Medicine, Staten Island University Hospital, Staten Island, USA; 2 Internal Medicine, Touro, Staten Island, USA; 3 Nephrology, Staten Island University Hospital, Staten Island, USA; 4 Cardiology, Staten Island University Hospital, Staten Island, USA

**Keywords:** acute coronary syndrome, red blood cell distribution width, contrast induced kidney injury

## Abstract

Background: Contrast-induced acute kidney injury (CI-AKI) is a relatively reversible cause of acute kidney injury (AKI) that occurs after radiocontrast media administration. It is a common complication after percutaneous coronary intervention, especially in patients with acute coronary syndrome (ACS). The aim of this study is to determine the utility of red cell distribution width (RDW) in predicting CI-AKI in patients with ACS and to determine the value of adding RDW to the Mehran risk score (MRS) on admission.

Methods: A total of 161 patients who presented with ST-elevation myocardial infarction (STEMI) or non-STEMI were identified retrospectively between January 2015 and December 2016. Patients were divided into two groups, those who developed CI-AKI after percutaneous coronary intervention (PCI) and those who did not.

Results: A total of 161 patients were analyzed. Of them, 12 developed CI-AKI (eight presented with STEMI and four presented with non-STEMI). RDW did not correlate with the development of CI-AKI (14.55 ± 1.48 vs 14.83 ± 1.21; p = 0.072). The areas under the receiver operating characteristic curves (ROCs) for RDW, MRS, and the combined model (MRS and RDW) for the prediction of CI-AKI were 0.721 (95% confidence interval (CI), 0.645 to 0.788; p=0.0024), 0.885 (95% CI, 0.825 to 0.930; p<0.0001), 0.890 (95% CI, 0.831 to 0.933; p<0.0001), respectively. Pairwise comparisons between ROCs for MRS vs the combined model yielded a non-significant p-value of 0.49. This signifies no added benefit for RDW to MRS for predicting CI-AKI.

Conclusion: RDW does not correlate with the development of CI-AKI in patients with ACS. The Mehran risk score remains a better indicator of CI-AKI risk assessment with no role for the addition of RDW to it. Further studies are needed to better assess predictors of CI-AKI in patients undergoing percutaneous coronary intervention.

## Introduction

Contrast-induced acute kidney injury (CI-AKI) is a well-recognized complication observed after the administration of iodinated contrast media during angiographic procedures [1]. It is defined as a greater than 25% increase in baseline serum creatinine or an absolute increase of 0.5 mg/dL within 48 hours after exposure and usually peaking within the following five days [1]. CI-AKI has been associated with adverse outcomes, including the need for renal replacement therapy, in-hospital complications, rehospitalization, increased length of stay, and mortality [2-3]. The need for contrast-based diagnostic and therapeutic cardiovascular procedures has been constantly increasing over the past decade. Furthermore, patients with acute coronary syndrome (ACS) have been observed to have nearly double the rates of CI-AKI [4-5]. Therefore, all patients undergoing percutaneous coronary angiography (PCI) should be evaluated for CI-AKI risk. At present, several risk scores have been developed. Of them, the Mehran risk score (MRS) remains the most widely used risk assessor for predicting CI-AKI risk [6]. In this retrospective study, we evaluated the predictive utility of red cell distribution width (RDW) for CI-AKI solely and when added to the Mehran risk score.

## Materials and methods

This is a single centered, retrospective cohort study done at a major community hospital in New York City and in one of the most diverse communities in the United States. Patients who presented to our hospital between January 2015 and December 2016 with a diagnosis of acute coronary syndrome (ACS) were identified. Only patients with ST-elevation myocardial infarction (STEMI) or non-STEMI who underwent PCI were included. A total of 161 patients were included and were divided into those who developed CI-AKI and those who didn’t. CI-AKI was defined as a greater than 25% increase in baseline serum creatinine or an absolute increase of 0.5 mg/dL within 48 hours after receiving contrast media for PCI. Patients with end-stage renal disease on hemodialysis, liver cirrhosis, autoimmune disease, active infection, and hematological malignancies were excluded. Demographics and laboratory characteristics were identified and analyzed on admission in both groups.

For continuous variables, the differences between the groups were estimated using the Wilcoxon two-sample test. For categorical variables, Fisher’s exact test was used. All statistical tests were two-sided and conducted at a 0.05 level of significance. A logistic regression analysis was performed to assess the predictive value of RDW. A receiver operating characteristic (ROC) curve analysis was performed to identify the optimal cut-off point for RDW, Mehran risk score, and the model at which sensitivity and specificity would be maximal for the prediction of CI-AKI. Areas under the curve (AUC) were calculated and a pairwise comparison of ROC curves was done.

## Results

**Figure 1:**
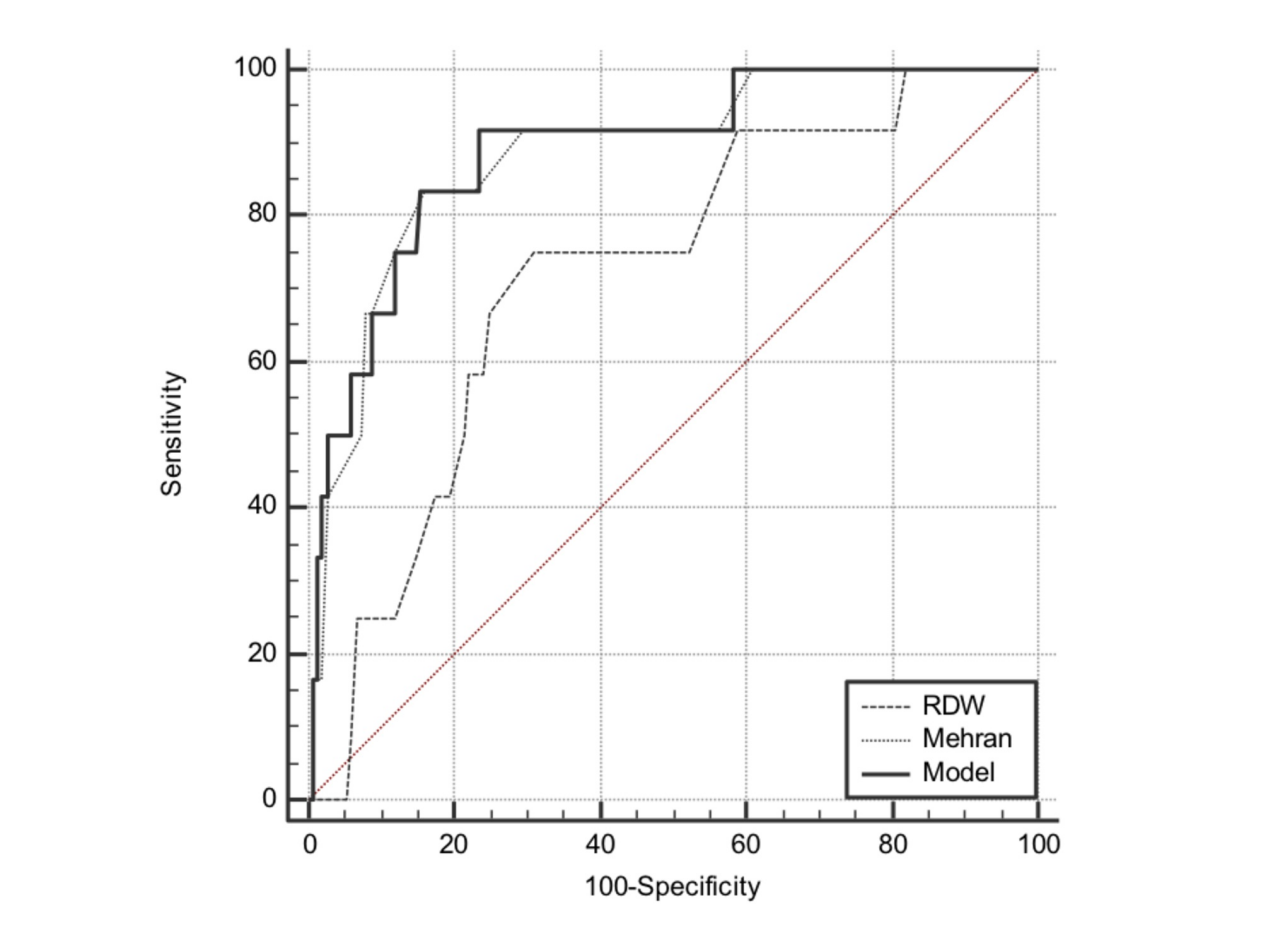
ROCs for RDW, MRS, and model with pairwise comparison between MRS and model giving a significance value (p=0.49) ROC: receiver operating characteristic; MRS: Mehran risk score

Of the 161 patients identified and analyzed, 149 didn’t have CI-AKI while 12 did (eight presented with STEMI and four presented with non-STEMI). Baseline characteristics for both groups are presented in Table 1. RDW didn’t correlate with the development of CI-AKI (14.55 ± 1.48 vs 14.83 ± 1.21; p = 0.072). MRS was predictive of CI-AKI in the included subjects. Congestive heart failure (CHF), diabetes mellitus (DM), use of an intra-aortic balloon pump (IABP), hemoglobin level (Hb), blood urea nitrogen (BUN), and the estimated glomerular filtration rate (GFR) were all significant predictors of CI-AKI and are all components of the Mehran risk score except blood urea nitrogen (BUN), as presented in Table 2. The logistic regression analysis failed to show any predictive value for RDW in CI-AKI (Table 3). The areas under the receiver operating characteristic curves (ROCs) for RDW, MRS, and the combined model (MRS and RDW) for the prediction of CI-AKI were 0.721 (95% confidence interval (CI), 0.645 to 0.788; p=0.0024), 0.885 (95% CI, 0.825 to 0.930; p<0.0001), and 0.890 (95% CI, 0.831 to 0.933; p<0.0001), respectively. A pairwise comparison of ROC curves failed to show any additional value for RDW to the Mehran risk score (p=0.49) (Figure 1).

**Table 1:** Baseline characteristics of the included patients Abbreviations: a: Fisher's exact test; b: Wilcoxon two-sample test; BMI: body mass index; CI-AKI: contrast-induced acute kidney injury; COPD: chronic obstructive pulmonary disease; IABP: intra-aortic balloon pump; PCI: percutaneous coronary intervention; PMHx: past medical history; STEMI: ST-elevation myocardial infarction

	No CI-AKI (149)	CI-AKI (12)	p-Value
Age (years)	62.5 ± 11	66.2 ± 11	0.26^b^
Male gender, n (%)	109 (73)	8 (66)	0.74^a^
Height (M)	1.7 ± 0.1	1.68 ± 0.06	0.39^b^
Weight (Kg)	86.8 ± 17.7	85 ± 10.6	0.84^ b^
BMI	29.88 ± 5.30	30.21 ± 4.61	0.77^ b^
STEMI, n (%)	72 (48)	8 (67)	0.37 ^a^
Non-STEMI, n (%)	76 (51)	4 (33)	0.37 ^a^
Hypertension, n (%)	103 (69)	11 (92)	0.18^ a^
Dyslipidemia, n (%)	92 (62)	9 (75)	0.54^ a^
COPD, n (%)	2 (1)	0 (0)	1.00^ a^
Prior PCI, n (%)	26 (17)	4 (33)	0.24^ a^
Congestive heart failure, n (%)	10 (6)	4 (33)	0.01^ a^
Diabetes mellitus, n (%)	31 (21)	7 (58)	0.01^ a^
No PMHx, n (%)	26 (17)	1 (8)	0.69^a^
IABP use, n (%)	1 (1)	2 (16)	0.01^a^
Amount of used contrast (ml)	142.72 ± 66.49	108.75 ± 112.94	0.11^b^
Hospital stay length (days)	5 ± 4	12 ± 18	0.24^ b^
In-hospital expiration, n (%)	2 (1)	5 (42)	<.0001^a^

**Table 2:** Laboratory parameters of the included patients Abbreviations: a: Fisher's exact test; b: Wilcoxon two-sample test; BUN: blood urea nitrogen; CI-AKI: contrast-induced acute kidney injury; eGFR: estimated glomerular filtration rate; MCV: mean corpuscular volume; N/L: neutrophil/lymphocyte; P/L: platelets/lymphocytes; RDW: red blood cell distribution width

	No CI-AKI (149)	CI-AKI (12)	p-value
Hemoglobin (g/dl)	14.13 ± 1.78	12.75 ± 2.49	0.01^b^
RDW (%)	14.035 ± 1.48	14.83 ± 1.21	0.07^b^
MCV (FL)	87.33 ± 6.7	84.83 ± 5.28	0.18^b^
Neutrophils count (10^3^ /µL)	6.50 ± 3.23	6.93 ± 2.64	0.70^b^
Platelets count (10^3^/µL)	241.22 ± 92.30	218.00 ± 79.76	0.40^b^
Lymphocytes count (10^9^/L)	2.67 ± 2.69	2.30 ± 1.03	0.64^ b^
N/L ratio	3.64 ± 4.10	2.95 ± 2.95	0.97^b^
P/L ratio	122.37 ± 73.26	111.29 ± 53.26	0.61^b^
BUN (mg/dl)	18.48 ± 9.64	29.58 ± 13.34	<0.001^b^
Creatinine (mg/dl)	1.08 ± 0.41	1.96 ± 0.61	<.0001^ b^
EGFR (ml/min/1.73 m)	72.42 ± 21.45	35.75 ± 16.91	<.0001^ b^
Creatinine within 48 hours (mg/dl)	1.02 ± 0.34	2.93 ± 0.82	<.0001^ b^
RDW (%)	14.55 ± 1.48	14.83 ± 1.21	0.07^b^
Mehran risk score	4.60 ± 3.51	11.33 ± 4.58	<.0001^ b^

**Table 3:** Logistic regression model for RDW and MRS Abbreviations: RDW: red blood cell distribution width; MRS: Mehran risk score

Effect	Odd Ratio	Confidence interval	p-Value
RDW	1.198	0.829-1.732	0.34
MRS	1.365	1.177-1.583	<.0001

## Discussion

Each year, more than 80-million iodinated contrast studies are performed across the globe. The trend towards minimally invasive diagnostic and interventional procedures that require the use of intravenous or intra-arterial contrast has been increasing [7]. With this increase, the incidence of CI-AKI has also been rising. In fact, cohort data have shown that CI-AKI is the third most common cause of AKI in patients admitted to the hospital [8]. Nevertheless, the exact pathophysiology of CI-AKI remains obscure, and several inflammatory mediators may play a role in oxidative stress and apoptosis.

In this study, we evaluated the role of red blood cell distribution width (RDW) as a predictive marker for CI-AKI. RDW is a numerical measure of red blood cell (RBC) volume variations. It is part of a standard complete blood count and is useful in eliciting the differential diagnosis of anemia. It has been associated with mortality in coronary artery disease (CAD) patients along with a higher rate of major cardiovascular adverse events [9-10]. The role of RDW in predicting contrast-induced acute kidney injury (CI-AKI) has been investigated previously. Mizuno et al. demonstrated that RDW was independently associated with CI-AKI and has an additional predictive value to the MRS [11]. Akkoyun et al. and Akin et al. also showed that RDW is an independent predictor for CI-AKI in STEMI patients undergoing PCI [12-13]. Kurtul et al. showed similar results but included patients with all types of ACS; however, more than 60% of them were STEMI patients [14]. The predictive utility for RDW is most likely explained by the fact that it signifies ongoing systemic inflammation [15]. Meanwhile, elevated RDW levels are consistent with increased anisocytosis among red blood cells, likely due to impaired erythrocyte maturation. Subsequently, this might indirectly reflect a state of systemic inflammation and constant exposure to oxidative stress and thus increased CI-AKI risk. However, in our analysis, this was not the case. This is most likely explained by the fact that we included patients with STEMI and NSTEMI as we believe CI-AKI prediction is more essential in patients with NSTEMI as there is some room for precautious measures prior to receiving contrast. Moreover, and since NSTEMI patients usually have more comorbidities at baseline than STEMI patients, we believe that other baseline characteristics are more important for CI-AKI prediction.

In summary, although RDW is a marker of inflammation that has a negative prognostic value in CAD and is linked to kidney dysfunction, the role of the usual predictive markers, such as heart failure and glomerular filtration rate (GFR), is more important. In our study, there was no significant difference in values between the two groups, and RDW didn’t provide an additional benefit to MRS. Therefore, we conclude that MRS remains the most useful predictive marker for CI-AKI.

## Conclusions

RDW does not correlate with the development of CI-AKI in patients with ACS. Mehran risk score remains a better indicator for CI-AKI risk assessment with no role for the addition of RDW to it. Further studies are needed to better assess predictors of CI-AKI in patients undergoing percutaneous coronary intervention.
